# Discordant pathological diagnosis of non‐alcoholic fatty liver disease: A prospective multicenter study

**DOI:** 10.1002/jgh3.12289

**Published:** 2019-12-17

**Authors:** Takuya Kuwashiro, Hirokazu Takahashi, Hideyuki Hyogo, Yuji Ogawa, Kento Imajo, Masato Yoneda, Takashi Nakahara, Satoshi Oeda, Kenichi Tanaka, Yuichiro Amano, Shinji Ogawa, Atsushi Kawaguchi, Shinichi Aishima, Masayoshi Kage, Kazuaki Chayama, Atsushi Nakajima, Yuichiro Eguchi

**Affiliations:** ^1^ Liver Center Saga University Hospital Saga Japan; ^2^ Division of Metabolism and Endocrinology, Faculty of Medicine Saga University Saga Japan; ^3^ Department of Gastroenterology and Hepatology JA Hiroshima General Hospital Hiroshima Japan; ^4^ Department of Gastroenterology and Hepatology Yokohama City University Graduate School of Medicine Yokohama Japan; ^5^ Department of Gastroenterology and Metabolism, Institute of Biomedical & Health Sciences Hiroshima University Hiroshima Japan; ^6^ Takeda Pharmaceutical Company, Ltd. Fujisawa Kanagawa Japan; ^7^ Section of Clinical Cooperation System, Center for Comprehensive Community Medicine, Faculty of Medicine Saga University Saga Japan; ^8^ Department of Pathology and Microbiology, Faculty of Medicine Saga University Saga Japan; ^9^ Kurume University Research Center for Innovative Cancer Therapy Kurume Japan

**Keywords:** elastography, noninvasive, observer error, reliability

## Abstract

**Background:**

Liver biopsy has been the standard procedure for diagnosing and evaluating the severity of non‐alcoholic fatty liver disease (NAFLD) and non‐alcoholic steatohepatitis (NASH); however, interobserver discordance remains a critical issue in its pathological diagnosis.

**Methods and Results:**

We examined the concordance rates of pathological scoring and diagnosis between pathologists at individual institutions (local diagnosis) and two central pathologists specialized in liver pathology (central diagnosis). A total of 150 patients with NAFLD underwent prospective liver biopsies. NAFLD activity score (NAS) and fibrosis stage were evaluated, and NASH was determined according to Matteoni's classification. NAS, scores for all NAS components, and fibrosis stage were diagnosed at a lower degree by central compared with local diagnosis. NASH was diagnosed in 34% of the patients according to central pathologists compared with 54% according to local pathologists (*P* < 0.001). The concordance rates for NAS, steatosis, inflammation, ballooning, fibrosis, and NASH diagnosis were 26.7, 62.7, 51.3, 48.7, 43.3, and 50.7%, respectively. The correlation coefficient between local and central diagnoses was the lowest for the scoring of ballooning (*ρ* = 0.218).

**Conclusion:**

Concordance rates among pathologists for the evaluation of NAFLD are currently poor, and simple and reliable diagnostic and evaluation criteria are urgently needed to improve the clinical management of NAFLD patients.

## Graphical abstract

We examined the concordance rates for pathological scoring and diagnosis of NAFLD between the pathologists The concordance rates for NAS, steatosis, inflammation, ballooning, fibrosis, and NASH diagnosis were 26.7%, 62.7%, 51.3%, 48.7%, 43.3%, and 50.7%, respectively. Concordance rates among pathologists for evaluation of NAFLD are currently poor, and simple and reliable diagnostic and evaluation criteria are urgently needed

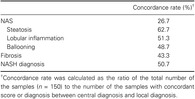

## Introduction

Non‐alcoholic fatty liver disease (NAFLD) is the most common cause of chronic liver disease worldwide and affects about 25% of the world population.^1^ NAFLD is classified as non‐alcoholic fatty liver (NAFL) or non‐alcoholic steatohepatitis (NASH), of which NASH is pathologically characterized by lobular inflammation and the presence of hepatocellular ballooning with or without fibrosis.^2^, ^3^ NAFLD increases overall and liver‐related mortality, with NASH considered to be a more progressive disease associated with a greater risk of liver cirrhosis and hepatocellular carcinoma than NAFL.^3^, ^4^, ^5^ Therefore, the differential diagnosis of NASH and NAFL has important implications in terms of the prognosis of NAFLD. However, recent studies demonstrated that fibrosis, rather than other histological features, was indicative of all‐cause and disease‐specific mortality in patients with NAFLD.^6^, ^7^, ^8^, ^9^ These studies therefore concluded that the severity of hepatic fibrosis was the most important pathological finding predicting the clinical outcome of NAFLD, rather than a diagnosis of NASH, which requires hepatocyte ballooning according to Matteoni's classification.^2^

Although histological diagnosis by liver biopsy remains the standard procedure for the diagnosis of NASH,^10^ liver biopsy is associated with numerous problems, including invasiveness, cost of diagnosis, sampling error, and diagnostic variation among observers.^11^, ^12^, ^13^, ^14^ However, numerous noninvasive biomarkers and procedures have recently been developed and evaluated for identifying NASH and determining the severity of hepatic fibrosis in patients with NAFLD.^15^ In this context, it is necessary to reconsider the significance of liver biopsy for the diagnosis and management of NAFLD and to evaluate the reliability of the pathological diagnosis of NAFLD/NASH and the assessment of its severity among pathologists. We therefore conducted a prospective multicenter study to compare the diagnostic performances of local and central pathologists for NAFLD.

## Methods

### *Patients*

A total of 176 consecutive patients with clinically and pathologically diagnosed NAFLD were enrolled from three institutions (Hiroshima University Hospital, Yokohama City University Hospital, and Saga University Hospital) between 2014 and 2016. This cohort was part of the clinical Comprehensive Analysis Study of NAFLD (COMPAS NAFLD; UMIN Clinical Trial Registry UMIN000013323). No patient had any etiology indicative of other liver diseases, including habitual alcohol intake (weekly ethanol consumption >140 g or daily ethanol consumption >20 g), hepatitis B surface antigen or hepatitis C antibody positivity, or abnormal serum thyroid hormone levels, and no patient had autoimmune liver disease, drug‐induced hepatotoxicity, hemochromatosis, or Wilson's disease. The protocol was approved by the clinical research ethics review committee of each facility. Each patient gave written informed consent to participate in the clinical study. The ethics committee of each participating institution approved this study, which was performed in accordance with the principles of the 1975 Declaration of Helsinki, revised in 2013.

### *Liver biopsy procedure and pathological evaluation*

**Figure 1:**
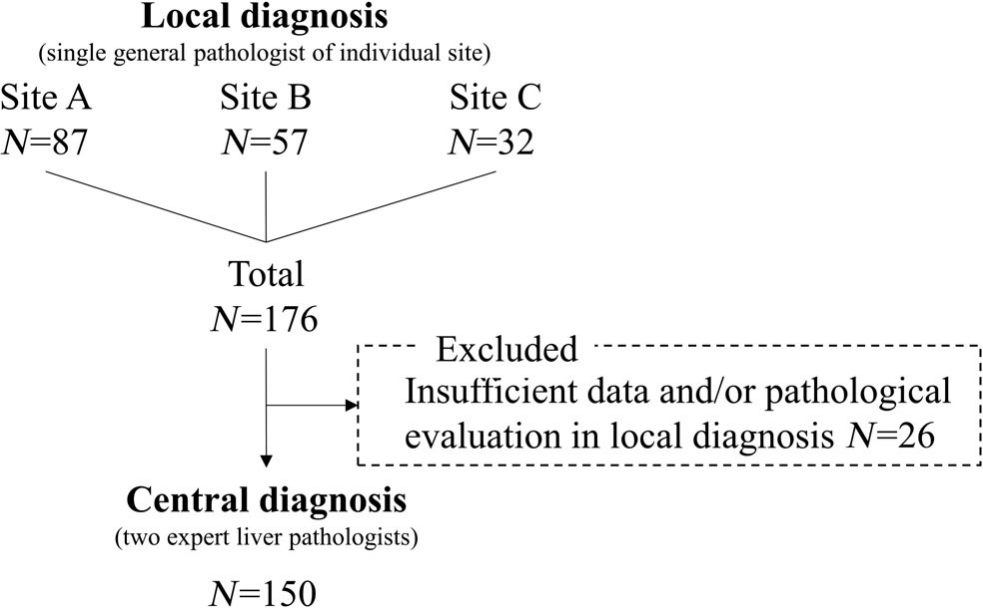
Study design. A single general pathologist evaluated liver biopsy in the individual site. All samples were collected and evaluated by the two expert central liver pathologists.

All patients underwent liver biopsy, and liver specimens were obtained percutaneously using a 16‐gauge biopsy needle. Biopsies were performed in the right lobe of the liver under ultrasound guidance. All liver biopsy samples were at least 20 mm long. The specimens were fixed in 10% formalin, paraffin embedded, sectioned, and stained with hematoxylin–eosin and Azan or Masson‐trichrome stain for histological evaluation. All liver biopsy specimens were evaluated by a single experienced general pathologist in the local institution, who was unaware of the clinical conditions and patient data and gave a local diagnosis. The same slides were also evaluated simultaneously by two different pathologists designated by our study group (S.A. and M.K.), who were experts in liver pathology and gave a consensual central diagnosis. All pathologists were unaware of the clinical information and were certified by the Japanese Society of Pathology. The samples were scored according to the NAFLD activity score (NAS).^16^ Lobular inflammation and steatosis were scored on a scale of 0–3 and ballooning on a scale of 0–2. Fibrosis stage was scored on a 5‐point scale (F0–F4) according to the Kleiner classification.^16^ NAFLD was defined as excessive fat accumulation in the liver with more than 5% of hepatocytes.^10^ NASH diagnosis was based on Matteoni's classification.^2^ In the 176 samples evaluated by the central pathologist, 26 samples were excluded because of insufficient data and/or pathological evaluation in local diagnosis. The scoring, classification, and NASH diagnosis were compared between the local diagnosis and central diagnosis (Fig. 1). Concordance rate was calculated as the ratio of the total number of the samples (*n* = 150) to the number of the samples with concordant score or diagnosis between the central diagnosis and local diagnosis.

### *Statistical analysis*

The local and central diagnostic assessments were compared using Wilcoxon's signed‐rank test, McNemar's test, and Spearman's rank correlation coefficient analysis. Agreement of ordered categorical variables was evaluated by weighted κ with linear weighting, which considers the proximity of categories.^17^ All statistical analyses were carried out using IBM SPSS Statistics ver.25 software (SPSS Japan, Tokyo, Japan).

## Results

### *Comparison of pathological scoring and staging of NAFLD between local and central pathologists*

**Figure 2:**
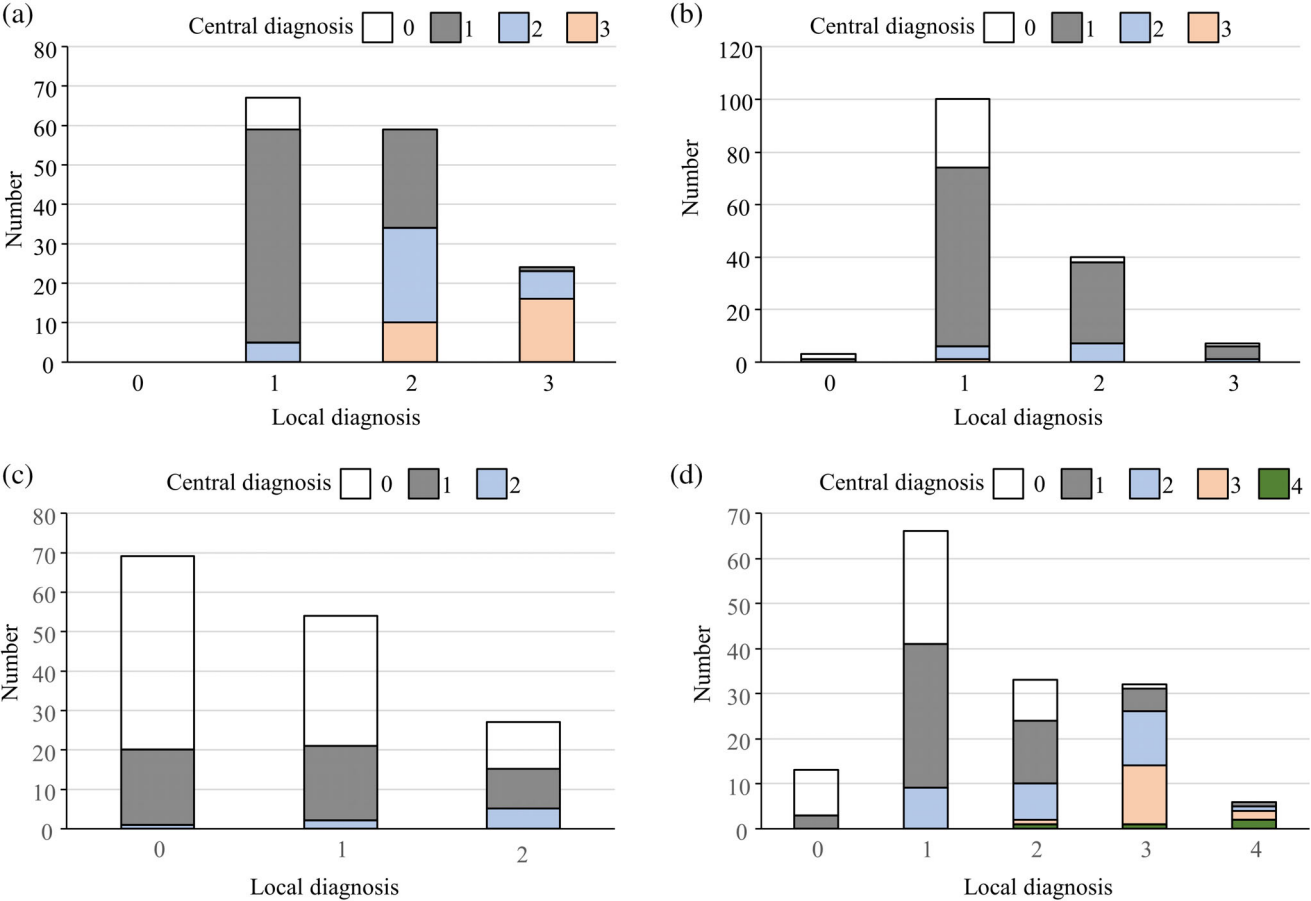
Discordance of pathological evaluation of NAFLD between local and central pathologists. (a) steatosis, (b) lobular inflammation, (c) ballooning, and (d) fibrosis

The pathological diagnoses made by the local and central pathologists are shown in Table 1 and Figure 2. NAS, individual components of NAS, and fibrosis stage were diagnosed at a significantly lower degree by the central, compared with the local, pathologists. Eighty‐one patients (54%) received a diagnosis of NASH based on Matteoni's classification, according to local diagnosis, compared with only 51 patients (34%) according to central diagnosis. Central pathologists diagnosed eight patients with non‐NAFLD according to Matteoni's classification.

**Table 1:** Comparison of pathological scoring and staging of NAFLD between local and central pathologists

	Local (*n* = 150)	Central (*n* = 150)	*P* value
NAS (0–4/5–8)	105/45	132/18	<0.001
Steatosis (0/1/2/3)	0/67/59/24	8/80/36/26	<0.001
Lobular inflammation (0/1/2/3)	3/100/40/7	31/105/13/1	<0.001
Ballooning (0/1/2)	69/54/27	94/48/8	<0.001
Fibrosis stage (F0/1/2/3/4)	13/66/33/32/6	45/55/30/16/4	<0.001
Matteoni's classification (non‐NAFLD/Type1‐2/Type3‐4)	0/69/81	8/91/51	<0.001†

† Comparison in distribution of NASH or non‐NASH by McNemar's test.

### *Concordance between local and central diagnoses*

The concordance rates for the diagnosis of steatosis, lobular inflammation, and ballooning according to NAS were 62.7, 51.3, and 48.7%, respectively (Table 2), with concordances of 26.7% for total NAS and 43.3% for fibrosis stage. The diagnosis concordance for distinguishing between NAFL and NASH was 50.7% according to Matteoni's classification. The κ score was also determined to evaluate diagnosis concordance (Table 3). Similar to the result of concordance rates, κ score for the diagnosis of ballooning (0.57) was lower than that for other components of NAS and fibrosis stage.

**Table 2:** Diagnosis concordance rate between local and central pathologists

	Concordance rate (%)†
NAS	26.7
Steatosis	62.7
Lobular inflammation	51.3
Ballooning	48.7
Fibrosis	43.3
NASH diagnosis	50.7

† Concordance rate was calculated as the ratio of the total number of the samples (*n* = 150) to the number of the samples with concordant score or diagnosis between central diagnosis and local diagnosis.

**Table 3:** Diagnosis agreement between local and central pathologists

Categories (diagnosis)	*κ* score
Steatosis (score 0–1 or score 2–3 in NAS)	0.79
Lobular inflammation (score 0–1 or score 2–3 in NAS)	0.70
Ballooning (score 0 or score 1–2 in NAS)	0.57
Fibrosis (stages 0–1 or stages 2–4 in Kleiner classification)	0.74
NASH diagnosis (non‐NASH or NASH in Matteoni's classification)	0.53

### *Correlation between local and central diagnoses*

**Figure 3:**
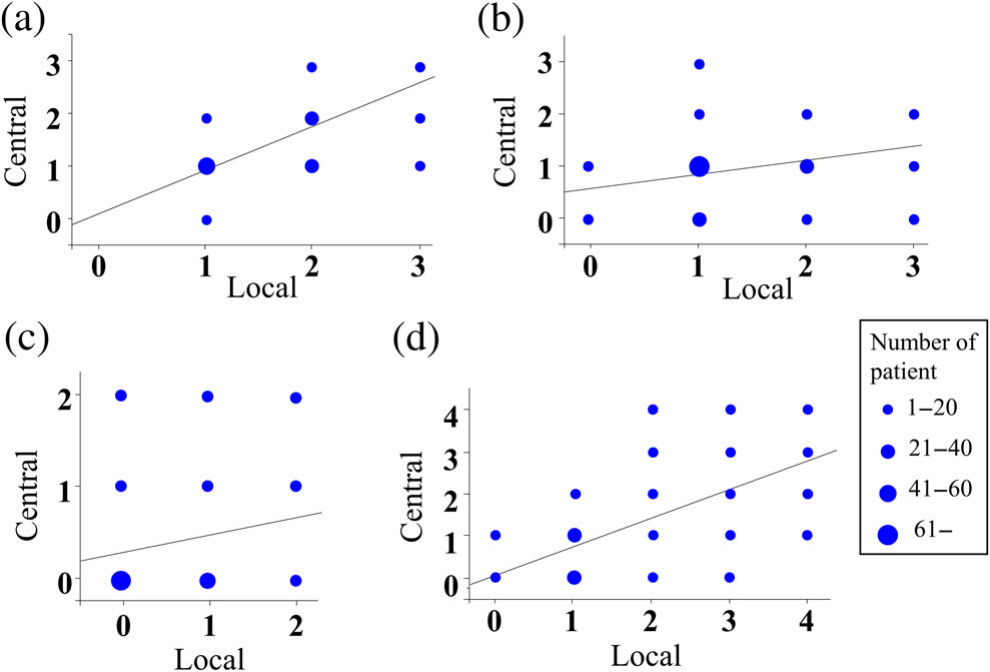
Correlation analysis between local and central diagnosis in (a) steatosis, (b) lobular inflammation, (c) hepatocyte ballooning, and (d) fibrosis. Dot size represents the number of patients in each score.

We tested the correlations between the local and central diagnoses and compared the correlation coefficients among the different pathological findings. There was a significant correlation between the local and central diagnoses for steatosis (*ρ* = 0.709, *P* < 0.0001) but lower correlation coefficients for inflammation (*ρ* = 0.286, *P* = 0.0005) and ballooning (*ρ* = 0.218, *P* = 0.0079) (Fig. 3a–c). Correlation for fibrosis stage was the most significant pathological finding (*ρ* = 0.627, *P* < 0.0001) (Fig. 3d).

## Discussion

The current study demonstrated a serious discordance between pathologists in the pathological diagnosis of NAFLD. Focusing on the individual pathological components, the lowest concordance (48.7%) and lowest correlation coefficient (*ρ* = 0.218) between local and central diagnoses was observed for the diagnosis of ballooning. These data suggest the existence of significant interobserver error in the diagnosis of hepatocyte ballooning, which is a key finding for the diagnosis of NASH.^2^, ^10^, ^16^ Local pathologists identified ballooning (grades 1–2) in 54% of patients, compared with only 37.3% according to central diagnosis, suggesting that pathologists not specialized in liver pathology might overdiagnose ballooning. The diagnosis of inflammation showed a similar trend, with 2% of patients diagnosed with grade 0 inflammation according to local diagnosis compared with almost 10 times more patients according to the central diagnosis (20.7%). These discordances indicated poor reliability for a diagnosis of NASH according to Matteoni's classification.

Hepatic fibrosis has been strongly implicated in the long‐term prognosis of NAFLD patients.^6^, ^7^, ^8^, ^9^ Moreover, NAFLD prognosis is independent of the diagnosis of NASH/non‐NASH.^7^, ^8^ It is therefore critical to identify patients at higher risk of NAFLD with advanced fibrosis in order to optimize their management. However, our results showed that the concordance rate for a diagnosis of fibrosis was only 43.3%. Moreover, local pathologists diagnosed stage 3 or 4 fibrosis in 25.3% of patients, compared with 13.3% by central pathologists. Previous studies showed that the liver‐related mortality rate increased exponentially in NAFLD patients with advanced liver fibrosis.^8^ An overdiagnosis of hepatic fibrosis would increase the number of patients with therapeutic indications, thus increasing the economic burden in light of the upcoming availability of novel therapeutic agents for fibrosis in NAFLD.

Numerous studies have investigated the interobserver reliability for pathological diagnosis. Theodossi *et al*. reported a concordance rate of final pathological diagnosis of 15% in various liver diseases.^17^ In chronic viral hepatitis, agreement of scoring and staging between the general pathologists and expert pathologists was not adequate, and the experience level of pathologists could affect the agreement of diagnosis.^18^, ^19^ In NAFLD, Younossi *et al*. identified that discrepancy in the diagnosis of inflammation among pathologists is more severe in comparison with fibrosis.^13^ Juluri *et al*. reported that agreement (*κ* score) between the diagnosis of two pathologists (community pathologist and expert pathologist) was 0.62 for steatosis, 0.44 for lobular inflammation, 0.25 for ballooning, 0.40 for NAS, 0.35 for fibrosis, and 0.46 for non‐NASH/NASH diagnosis,^20^ suggesting that interobserver reliability was the highest in the diagnosis of steatosis and lowest in the diagnosis of ballooning. Our study confirmed the findings of Juluri *et al*. that agreement on the diagnosis of ballooning is the most difficult to obtain. In contrast, however, the κ score for the diagnosis of fibrosis stage in the study by Juluri *et al*. was only 0.35 and was lower than for other pathological diagnoses, whereas our study showed better agreement (*κ* = 0.76 in Table 2). Gawrieh *et al*. compared the diagnosis of one senior pathologist with that of one junior pathologist and demonstrated *κ* = 0.72 for the diagnosis of steatosis, *κ* = 0.64 for the diagnosis of fibrosis stage, and *κ* = 0.32 for the diagnosis of ballooning.^14^ According to this evidence and the results of our study, concordance in the diagnosis of ballooning tends to be low for NAFLD, whereas concordance in the diagnosis of fibrosis stage varies among studies. Moreover, differences in experience between the pathologists could be a factor that affects concordance. Indeed, training and prior consent of scoring and diagnosis, including definitions of detailed morphological criteria, increase the concordance of diagnosis and scoring by pathologists.^14^, ^21^

The κ score obtained in the current study was 0.53–0.79 for the comparison between central diagnosis and local diagnoses, which could be interpreted as “moderate” or “good.”^22^ However, there are several limitations in an evaluation of interobserver reliability using the κ score. It was reported that prevalence bias could affect κ score.^23^ For example, a significant difference in the prevalence among the categories could result in either a significantly high or low κ score. Moreover, because the scoring is quantitative, a weighted κ score, which generally tends to be higher than a nonweighted score, should be statistically used as we did in the current study; however, in terms of clinical significance, a difference of 1 point in the scoring system creates a serious discrepancy. For example, the difference between a ballooning score of 0 and 1 could result in a diagnosis of non‐NASH or NASH. Therefore, the nonweighted κ score could also be referenced for the evaluation of concordance of pathological diagnosis to determine the difference between clinically important scores. Indeed, concordance was poor to moderate if a nonweighted κ score was used in the current study (*κ* = 0.59 for steatosis score 0–1 or 2–3, *κ* = 0.14 for lobular inflammation score 0–1 or 2–3, *κ* = 0.15 for ballooning score 0 or 1–2, and *κ* = 0.47 for fibrosis stage 0–1 or 2–4; data not shown). Taken together, because the *κ* score could mislead the interpretation of agreement, the results of simple concordance/discordance rate, correlation coefficient, and clinical significance should be carefully considered for fuller comprehension of agreement.^23^

Liver biopsy remains the gold standard for characterizing changes in liver histology in patients with NAFLD; however, liver biopsy has some limitations, including its cost, the risks of morbidity and (rarely) mortality, and the need for adequate experience to provide a pathological diagnosis.^10^ It has therefore been considered that liver biopsy should only be performed in patients most likely to benefit from the diagnosis, therapeutic guidance, and prognostic information.^24^ However, the pathological diagnosis of NAFLD should now be reconsidered in terms of NASH, which does not affect the clinical outcome, and in terms of the evaluation of histological fibrosis, which is the most important finding for predicting mortality risk in NAFLD. Novel reliable screening and diagnostic strategies based on the evaluation of NAFLD and fibrosis, other than liver biopsy, are thus required to identify NAFLD patients at significant risk of mortality.^25^

Recent research into noninvasive biomarkers for detecting hepatic fibrosis in NAFLD may affect the clinical significance of liver biopsy. Serum biomarkers, including keratin 18,^26^, ^27^ type III procollagen peptide,^28^ and type III collagen propeptide,^29^ as well as noninvasive scoring systems, including the NAFLD fibrosis score,^30^ Fibrosis‐4 index,^31^ AST/platelet ratio index,^32^ FibroMeter,^33^, ^34^ and BARD score,^35^ have demonstrated high diagnostic accuracy and reliability for evaluating liver fibrosis and diagnosing NASH. Ultrasound, including vibration‐controlled transient elastography (VCTE), has also been well studied and has been used clinically to predict liver fibrosis in NAFLD. Two recent studies demonstrated the excellent diagnostic performance of VCTE in patients with biopsy‐proven NAFLD.^36^, ^37^ Furthermore, the imaging‐based magnetic resonance (MR) technique, MR elastography, showed greater diagnostic accuracy than VCTE for the prediction of liver fibrosis in patients with NAFLD,^38^, ^39^ as recently confirmed in a multicenter study.^40^ As an alternative to liver biopsy, MR elastography could serve as a new gold standard for the assessment of liver fibrosis with NAFLD.^41^ The rate of agreement on fibrosis stage between different radiologists reading MR elastography has been shown to be greater than that of separate pathologists assessing biopsy specimens.^32^, ^33^, ^42^, ^43^, ^44^ Therefore, in our opinion, MR elastography will become a new gold standard and benchmark, which should be used to evaluate the utility of biomarkers. MR imaging can also be used for the noninvasive quantification of liver steatosis by spectroscopy^45^ or by measuring the proton density fat fraction,^46^, ^47^ as widely used in NAFLD clinical trials.^48^ Overall, therefore, several noninvasive procedures are available for predicting liver fibrosis in NAFLD. Although further studies are needed to determine whether these procedures predict clinical outcomes, including mortality, in patients with NAFLD, they may provide alternatives to liver biopsy in clinical practice.

In conclusion, there is significant discordance among pathologists in relation to the diagnosis of NASH and in NAS scoring. Discordances in the diagnosis of ballooning and fibrosis are critical for the diagnosis of NASH and the management of NAFLD, respectively. There is therefore an urgent need for globally agreed, simple pathological diagnostic criteria for NAFLD or the establishment of alternative noninvasive and quantitative methods to provide diagnostic and reference information for the clinical management of NAFLD patients.
